# Functional analysis of Normalized Difference Vegetation Index curves reveals overwinter mule deer survival is driven by both spring and autumn phenology

**DOI:** 10.1098/rstb.2013.0196

**Published:** 2014-05-26

**Authors:** Mark A. Hurley, Mark Hebblewhite, Jean-Michel Gaillard, Stéphane Dray, Kyle A. Taylor, W. K. Smith, Pete Zager, Christophe Bonenfant

**Affiliations:** 1 Idaho Department of Fish and Game, Salmon, ID, USA; 2 Wildlife Biology Program, Department of Ecosystem and Conservation Sciences, University of Montana, Missoula, MT, USA; 3 Department of Biodiversity and Molecular Ecology, Research and Innovation Centre, Fondazione Edmund Mach, San Michele all'Adige, Trentio, Italy; 4 UMR CNRS 5558, Laboratoire Biométrie et Biologie Évolutive, Université Claude Bernard, Lyon 1, 43 boulevard du 11 novembre 1918, 69622 Villeurbanne Cedex, France; 5 Department of Botany, University of Wyoming, Laramie, WY, USA; 6 Numerical Terradynamics Simulation Group, Department of Ecosystem and Conservation Sciences, University of Montana, Missoula, MT, USA; 7 Idaho Department of Fish and Game, Lewiston, ID, USA

**Keywords:** demography, Normalized Difference Vegetation Index, phenology curve, population dynamics, ungulate, winter severity

## Abstract

Large herbivore populations respond strongly to remotely sensed measures of primary productivity. Whereas most studies in seasonal environments have focused on the effects of spring plant phenology on juvenile survival, recent studies demonstrated that autumn nutrition also plays a crucial role. We tested for both direct and indirect (through body mass) effects of spring and autumn phenology on winter survival of 2315 mule deer fawns across a wide range of environmental conditions in Idaho, USA. We first performed a functional analysis that identified spring and autumn as the key periods for structuring the among-population and among-year variation of primary production (approximated from 1 km Advanced Very High Resolution Radiometer Normalized Difference Vegetation Index (NDVI)) along the growing season. A path analysis showed that early winter precipitation and direct and indirect effects of spring and autumn NDVI functional components accounted for 45% of observed variation in overwinter survival. The effect size of autumn phenology on body mass was about twice that of spring phenology, while direct effects of phenology on survival were similar between spring and autumn. We demonstrate that the effects of plant phenology vary across ecosystems, and that in semi-arid systems, autumn may be more important than spring for overwinter survival.

## 1. Introduction

A major challenge for the application of remote sensing to monitoring biodiversity responses to environmental change is connecting remote sensing data to large-scale field ecological data on animal and plant populations and communities [1]. Large herbivores, for example ungulates, are an economically and ecologically important group of species [2] with a global distribution and varied life-history responses to climate that are very sensitive to the timing and duration of plant growing seasons [3]. Until recently, monitoring plant phenology and the nutritional influences on ungulate life histories have been impossible at large spatial scales owing to the intense effort necessary to estimate even localized plant phenology. The remote sensing community has largely solved this issue by partnering with ecologists to provide circumpolar remotely sensed vegetation indices, fuelling the recent explosion of the integration of remote sensing data into wildlife research and conservation [1,4,5]. With satellites such as the Advanced Very High Resolution Radiometer (AVHRR), the Moderate Resolution Imaging Spectroradiometer (MODIS), the Satellite Pour l'Observation de la Terre (SPOT) [6,7], and growing tool sets for ecologists [8], derived metrics are being commonly used to analyse the ecological processes driving wildlife distribution and abundance [5]. Indices such as the Normalized Difference Vegetation Index (NDVI) and the enhanced vegetation index (EVI) strongly correlate with vegetation productivity, track growing season dynamics [9,10] and differences between landcover types at moderate resolutions over broad spatio-temporal scales [6]. Indices extracted from NDVI correlate with forage quality and quantity [5,11,12] and thus have become invaluable for indexing habitat quality for a variety of ungulates [11,13,14]. For example, only this technology can track a landscape scale plant growth stage that ungulates often select to maximize forage quality [15]. Because of this spatial and temporal link to forage quality, NDVI can be predictive of ungulate nutritional status [11], home range size [16], migration and movements [12,14,17]. An increasing number of studies have also linked NDVI to body mass and demography of a wider array of vertebrates. While there have been recent reviews of the link between NDVI and animal ecology [5], few provided examples where autumn phenology was considered. We conducted a brief review of recent studies to expose readers working at the interface of remote sensing and biodiversity conservation to the pre-eminent focus on spring phenology using *a priori* defined variables. From the literature review we performed, 16 out of 22 case studies in temperate areas focused on spring, while three used a growing season average, and only three considered both spring and autumn phenology (table 1). Most studies were based on NDVI metrics describing the active vegetation period, such as start, end and duration of growing season (table 1). Moreover, all but one (see table 1, [40]) was based on *a priori* defined NDVI metrics assumed to provide a reliable description of plant phenology through the growing season. From this empirical evidence so far reported (see table 1 for details), spring phenology appears as an important period in temperate systems. However, recent field studies on ungulates emphasized the critical importance of late summer and autumn nutritional ecology, suggesting vegetation conditions during this period will also influence population performance of large herbivores. Our brief review complements that of Pettorelli *et al*. [5] and illustrates the importance of considering phenological dynamics over the entire growing season.

**Table 1.** A brief literature survey of the studies that investigated relationships between NDVI metrics and life-history traits linked to performance and population abundance. The literature survey was performed using ISI web of knowledge using the keywords ‘NDVI and survival’, ‘NDVI and body mass’, ‘NDVI and body weight’, ‘NDVI and reproductive success’, ‘NDVI and recruitment’, ‘NDVI and population growth’ and ‘NDVI and population density’. Only studies performed on vertebrate species were retained. For each case study, the table displays the focal trait(s), the focal species, the NDVI metric(s) used, the outcome (‘+’: positive association between NDVI and performance; ‘−’: negative association between NDVI and performance; ‘0’: no statistically significant association between NDVI and performance), the reference and the location of the study.

trait	species	NDVI metrics	outcome	location	references
protein mass body mass carcass mass body fat	caribou *Rangifer tarandus*	average NDVI in June	protein mass: + other traits: 0	Québec-Labrador (Canada)	[18]
birth mass juvenile autumn mass	caribou *Rangifer tarandus*	average NDVI in June	+	Québec-Labrador (Canada)	[19]
population density juvenile body mass	semi-domesticated reindeer *Rangifer tarandus*	summed NDVI over the breeding season	juvenile mass: 0 population density: + (in populations with poor winter ranges only)	Norway (across populations)	[20]
population size	lesser grey shrike *Lanius minor*	NDVI in May–June (breeding areas) NDVI in January–March (wintering areas)	+	France Spain (breeding areas) Kalahari (wintering areas)	[21]
reproductive performance (lamb/ewe in December)	sheep *Ovis aries*	NDVI in March–May NDVI in May	NDVI in May: + NDVI in March–May: 0	northwestern Patagonia	[22]
survival	African elephant *Loxodonta africana*	seasonal maximum NDVI	juvenile survival: + adult survival: 0	Kenya	[23]
parasite loading	red-legged partridge *Alectoris rufa*	yearly mean NDVI	+	Spain	[24]
body mass	red deer *Cervus elaphus*	monthly NDVI	spring NDVI: + (Spain only) other metrics/populations: 0	Europe (across population)	[25]
wing length tail length clutch size body mass (males and females)	barn swallow *Hirundo rustica*	NDVI in December–February (wintering areas)	male wing length, male and female tail length, clutch size: + other traits: 0	Italy (breeding area) Africa (wintering areas)	[26]
juvenile and adult survival	white stork *Ciconia ciconia*	NDVI in October–November (Sahel) NDVI in December–February (eastern and southern Africa)	+	eastern Germany Poland (breeding areas)	[27]
adult survival	barn swallow *Hirundo rustica*	NDVI in September–November NDVI in December–February NDVI in March–May (wintering areas in Africa)	+	Denmark	[28]
conception rates	African elephant *Loxodonta africana*	seasonal NDVI (wet versus dry seasons)	+	Kenya	[29,30]
juvenile and adult survival	Egyptian vulture *Neophora percnopterus*	yearly NDVI (wintering areas) NDVI in September–June (breeding areas)	+	Spain	[31]
survival reproductive success	red-backed shrike *Lanius collurio*	NDVI in September–October (Sahel) NDVI in December–March (South Africa) NDVI in April (Germany)	survival: + (NDVI in December to March) reproductive success: + (NDVI in September–October)	Germany	[32]
juvenile survival	greater sage grouse *Centrocercus urophasianus*	NDVI in May–August NDVI and Max NDVI in May, June, July and August	+ (trends only) strong covariation among NDVI metrics	Idaho Utah (USA)	[33]
body mass	red deer *C. elaphus*	NDVI in the 1st of May	+	Norway	[34]
juvenile body mass	roe deer *Capreolus capreolus*	summed NDVI in April–May summed NDVI in August–October	+ (Chizé population) 0 (Trois Fontaines population)	France	[35]
kidney mass	hystricognath rodents	yearly NDVI (calculated from monthly NDVI)	−	South America (across species	[36]
body mass	moose *Alces alces*	seven NDVI metrics (PCA)	+	Norway	[37]
body mass	wild boar *Sus scrofa* roe deer *Capreolus capreolus*	summed NDVI over the growing season	roe deer: 0 wild boar: 0	Poland	[38]
body condition	raccoon dog *Nyctereutes procyonoides*	four NDVI metrics (onset of spring, peak NDVI, summed NDVI over the growing season and rate of NDVI increase in spring)	onset of spring: − peak NDVI and summed NDVI: + rate of NDVI increase: 0	Finland	[39]
juvenile body mass reproductive success	reindeer *Rangifer tarandus*	EVI modelled using a double logistic function. Use of the parameters S (onset of spring), mS (rate of EVI increase) and mEVI (plant productivity)	S and mEVI on both mass and reproductive success: +	Norway	[40]
juvenile body mass pregnancy rate	elk *C. elaphus*	NDVI correlated with bi-weekly forage biomass and quality over the previous growing season	exposure to higher predicted forage quality: + juvenile body mass + female pregnancy	Canada	[14]
juvenile mass	sheep *Ovis aries* (two breeds)	NDVI in late May summed NDVI in June–August	NDVI in late May: + summed NDVI in June–August: 0 or – depending on the breed	Norway	[41,42]
population size	common house-martin *Delichon urbicum* common swift *Apus apus*	NDVI in December–February (wintering areas in Africa)	+	Italy	[43]
juvenile body mass	chamois *Rupicapra rupicapra*	five NDVI metrics (NDVI slope in spring, NDVI maximum slope in spring, maximum NDVI, date of NDVI peak, summed NDVI in March)	+ (summed NDVI in March the best predictor)	France	[44]
juvenile growth juvenile survival	mountain goat *Oreamnos americana* bighorn sheep Ovis *canadensis* alpine ibex *Capra ibex*	summed NDVI in May summed NDVI over the growing season rate of NDVI change	rate of NDVI change: − other metrics: 0	Canada Italy	[45]
population abundance	American redstarts *Setophage ruticilla*	NDVI in December–March (wintering areas)	+	North America (breeding areas) Carribean – Cuba (wintering areas)	[46]
reproductive success survival	white-tailed deer *Odocoileus virginianus*	summed NDVI in May–August rate of NDVI change between May and June maximum change between May and June	summed NDVI in May–August on reproductive success: + rate of NDVI change and maximum change on reproductive success: − effects on survival: 0	Anticosti, Québec (Canada)	[47]
population density	murine rodent *Akodon azarae*	seasonal NDVI	+	Argentina	[48]
population rate of increase	kangaroos *Macropus* sp.	NDVI for six and 12 months	+ (but not better predictor than rainfall)	Australia	[49]

Despite this focus on spring phenology, the best existing approach is to use a number of standardized growing season parameters derived from NDVI describing the onset, peak and cessation of plant growth. Unfortunately, these useful parameters are often highly correlated. In Wyoming for example, the start of the growing season was delayed and the rate of green-up was slower than average following winters with high snow cover [50], but these ecologically different processes were highly correlated. Thus, an important barrier to understanding the complex influence of growing season dynamics on ungulate survival is how to disentangle correlated plant phenology metrics. Another underappreciated barrier is the challenge of harnessing the time-series nature of NDVI data, which requires specific statistical tools; no previous study has attempted to describe how the NDVI function varies across an entire growing season or discriminates between sites. To fill this important gap, the joint use of functional analysis [51] to characterize seasonal variation in NDVI curves and path analyses [52] to assess both direct and indirect effects of plant phenology offers a powerful way to address entangled relationships of plant quality and their effects on population dynamics of ungulates.

Pioneering experimental work on elk (*Cervus elaphus*) [53] has led to a growing recognition that in temperate areas, late summer and autumn nutrition are important drivers of overwinter survival and demography of large herbivores [53,54]. Summer nutrition first affects adult female body condition [54], which predicts pregnancy rates [53–55], overwinter adult survival rates [54,56], litter size [57] as well as birth mass and early juvenile survival [57–59]. The addition of lactation during summer increases nutritional demand and thus is an important component of the annual nutritional cycle [47,60]. Nutrition during winter (energy) minimizes body fat loss [58], but rarely changes the importance of late summer and autumn nutrition for survival of both juveniles and adults [53]. Winter severity then interacts with body condition to shape winter survival of ungulates [54,61] and can, in severe winters, overwhelm the effect of summer/autumn nutrition through increased energy expenditure, driving overwinter survival of juveniles.

Like most other large herbivores of temperate and northern areas, mule deer (*Odocoileus hemionus*) population growth is more sensitive to change in adult female survival than to equivalent change in other demographic parameters. Survival of adult female mule deer, however, tends to vary little [62,63]; see [64] for a general discussion. By contrast, juvenile survival shows the widest temporal variation, often in response to variation in weather [65–67] and population density [68]. This large variation in juvenile survival, especially over winter, often drives population growth of mule deer [58,62,63]. Fawns accumulate less fat than adults during the summer, which increases their mortality because variation in late summer nutrition interacts with winter severity [62,69]. While previous studies have shown that spring plant phenology correlates with early juvenile survival in ungulates, summer survival is not necessarily more important than overwinter survival. Yet, to date, the effect of changes in autumn plant phenology on overwinter juvenile survival remains unexplored.

Our first goal was to identify the annual variation of plant primary production and phenology among mule deer population summer range, measured using NDVI curves of the growing season. Second, with annual plant phenology characterized, we assessed both direct and indirect (through fawn body mass) effects of these key periods on overwinter survival of mule deer fawns. We used a uniquely long-term (1998–2011) and large-scale dataset to disentangle plant phenology effects on mule deer survival, encompassing 13 different populations spread over the entire southern half of Idaho, USA, while most previous studies have focused only within one or two populations. These populations represent diversity of elevations, habitat quality and climatological influences. We focused on overwinter fawn survival because previous studies [62,63] have demonstrated that this parameter is the primary driver of population growth.

However, the influences of plant phenology during the growing season and of winter severity on winter survival are not independent because they both involve a strong indirect effect of body mass. Mysterud *et al.* [70] used a path analysis to separate independent effects of summer versus winter on body mass. We present a novel methodological framework in which we analyse NDVI measurements using functional principal component analysis (FPCA) to discriminate among study areas in Idaho with differing autumn and spring phenology. We then use hierarchical Bayesian path analysis to identify factors of overwinter mule deer survival. Based on previous studies, we expected that plant phenology should be strongly associated with body mass of mule deer at six months of age, and that body mass and winter severity should interact to determine overwinter survival. We expected direct effects of plant phenology on winter survival to be weaker than winter severity because severe conditions may overwhelm nutritional improvements to fawn quality. We also expected early winter severity would affect overwinter fawn survival more than late winter severity [71].

## 2. Material and methods

### (a) Study areas

The study area spanned approximately 160 000 km^2^, representing nearly the entire range of climatic conditions and primary productivity of mule deer in Idaho. We focused on 13 populations with winter ranges corresponding to 13 Idaho game management units (GMUs); hereafter, we use GMU synonymous with population (figure 2). There are three main habitat types (called ecotypes hereafter) based on the dominant overstory canopy species on summer range: coniferous forests, shrub-steppe and aspen woodlands. The populations were distributed among the ecotypes (figure 2) with five populations in conifer ecotype (GMUs 32, 33, 36B, 39, 60A), two in shrub-steppe ecotype (GMUs 54, 58) and six in aspen (GMUs 56, 67, 69, 72, 73A, 76). Elevation and topographic gradients within GMUs affect snow depths and temperature in winter, and precipitation and growing season length in the summer, with elevation increasing from the southwest to the northeast. Conifer GMUs ranged in elevation from 1001 to 1928 m, but most were less than 1450 m. Winter precipitation (winter severity) varied widely (from 10 to 371 mm) in coniferous GMUs. Coniferous ecotype summer ranges are dominated by conifer species interspersed with cool season grasslands, sagebrush and understory of forest shrubs. Shrub-steppe GMUs ranged from 1545 to 2105 m, with winter precipitation from 24 to 105 mm. Summer range within shrub-steppe ecotypes was dominated by mesic shrubs (bitterbrush (*Purshia tridentata*), sagebrush (*Artemisia* spp.), rabbitbrush (*Chrysothamnus* spp.), etc.). Aspen ecotype GMUs were located in the east and south with winter use areas ranging from 1582 to 2011 m, with five of the six GMUs above 1700 m with early winter precipitation ranging from 25 to 146 mm. In summer, productive mesic aspen (*Populus tremuloides*) woodlands were interspersed with mesic shrubs.

### (b) Mule deer monitoring

**Figure 1.**
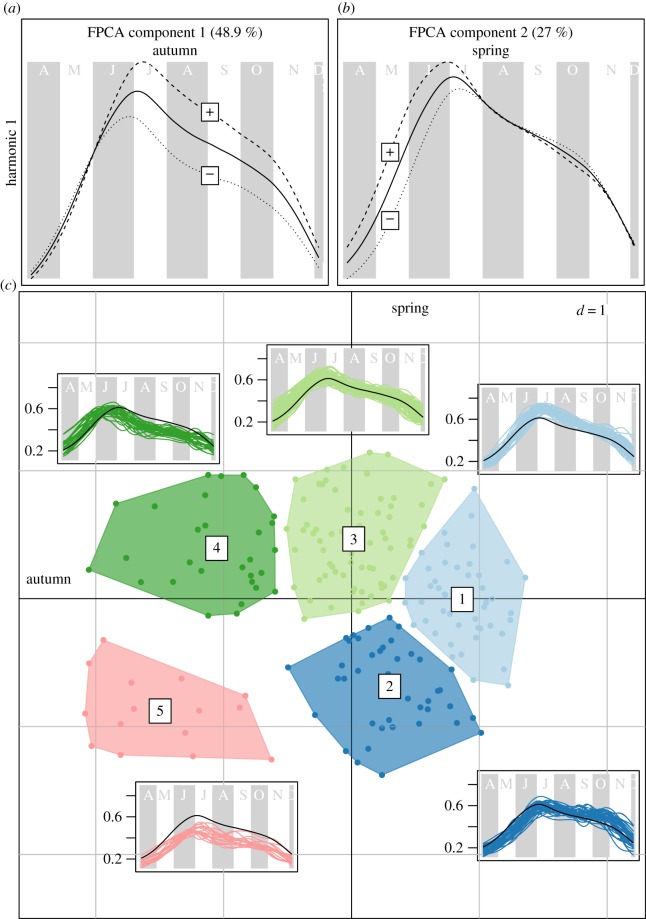
Results of FPCA of the typology of NDVI curves in Idaho, USA, from 1998 to 2011, from April (A) to November (N) for each population-year (dot) identifying two key periods, the spring (second FPCA component, the *Y*-axis) and the autumn components (first FPCA component, *X*-axis). (*a*) Variation in NDVI curves among populations and years was best explained by FPCA 1, which explained 48.9% of the variation and characterized primary production from June to October (e.g. summer/autumn). (*b*) FPCA 2 (*Y*-axis) characterized primary production in May and June and explained 27% of the seasonal variation. (*c*) NDVI typology was best characterized by five clusters, shown in different colours, that corresponded to different patterns of spring and autumn primary production, compared to the mean NDVI curve across all of Idaho. For example, typology 5 was characterized by low NDVI intensity in both spring and autumn, typology 3 by high NDVI intensity in both spring and autumn and typology 4 by high NDVI intensity in spring, but low in autumn, etc.

We radiocollared mule deer fawns at six months of age in the 13 GMUs (figure 1), resulting in 2315 mule deer fawns from 1998 to 2011. We captured fawns primarily using helicopters to move deer into drive nets [72], but occasionally by helicopter netgun [73] or clover traps [74]. Fawns were physically restrained and blindfolded during processing with an average handling time of less than 6 min. We measured fawn mass to the nearest 0.4 kg with a calibrated spring scale. Collars weighed 320–400 g (less than 2% of deer mass) were equipped with mortality sensors and fastened with temporary attachment plates or surgical tubing, allowing the collars to fall off the animals after approximately 8–10 months. We monitored between 20 and 34 mule deer fawns in each study area for a total of 185–253 annually from 1998 to 2011.

We monitored fawns with telemetry for mortality from the ground every 2 days between capture and 15 May through 2006, and then once at the first of each month during 2007–2011. We located missing fawns aerially when not found during ground monitoring. When a mortality signal was detected, we determined cause of death using a standard protocol [75]. In addition, we kept a minimal annual sample of approximately 600 adult females with radiocollars, using the same capture techniques as fawns. We used the composite sample of monthly aerial and mortality locations over the entire study period from these deer to estimate mule deer population ranges.

### (c) Defining population ranges of mule deer

We used the mule deer winter and summer ranges for each GMU as the main spatial units of analysis, and we extracted NDVI data from summer range and winter weather from winter range for each year from each population. We combined relocation points for all individuals and years in a single study site to estimate a 95% adaptive kernel home range for both summer and winter [76] for mule deer captured within a population. All deer populations were migratory with an average winter range size of 430 km^2^ and average summer range size of 3360 km^2^. Migratory periods, 1 April to 1 June and 1 October to 15 November, were excluded from the home range estimates, and remaining animal locations between 1 June and 30 September were used for summer, 1 December to 31 March for winter. Climate and habitat information was then summarized by the aggregate home range of radiocollared deer for winter and summer within each population.

### (d) Functional analysis of Normalized Difference Vegetation Index curves

We measured growing season phenology for each population-year using 1 km resolution, 7 day composite AVHRR NDVI data obtained from the National Oceanic and Atmospheric Administration (NOAA)-14, -16 and -17 AVHRR, and maintained by the United States Geological Survey (USGS; http://phenology.cr.usgs.gov/index.php) [77]. AVHRR NDVI data extend over the full temporal extent of our mule deer monitoring effort and has been shown to correspond well with MODIS NDVI data [77]. Radiometric sensor anomalies, atmospheric effects and geometric registration accuracies were previously accounted for according to [77]. Further, the data were accompanied by a cloud contamination mask, which was generated using an adaptation of the cloud clearing of AVHRR data (CLAVR) algorithm [76]. We then rescaled the processed data from the USGS 0-200 classification, with 100 corresponding to vegetated/non-vegetated threshold to the standard NDVI scale of −1 to 1. All cloud contaminated pixels were thus removed by applying this previously generated cloud contamination mask, and the resulting data gaps were infilled using a simple temporal interpolation method [10]. Finally, a minimum NDVI threshold value of zero was applied to define periods of little to no photosynthetic activity and filter any pixels containing ice and snow from the analysis. As phenological changes in NDVI only directly represent ungulate forage dynamics in non-forested vegetation types, we extracted NDVI values from only grass and shrub vegetation types (not burned within 5 years), which we characterized using SAGEMAP landcover data (2005 USGS, Forest and Rangeland Ecosystem Science Center, Snake River Field Station, Boise, ID, USA). Masking in this fashion directly parallels nutritional ecology as mule deer are adapted to feeding in open vegetation types and actively select these types during the growing season [78–80]. To encompass the entire growing season for each population-year, but excluding winter anomalies caused by varying snow condition, we restricted NDVI data to 15 March to 15 November. This time period provided a standardized measure of growing season while capturing the variability both within and between populations for comparing curves.

We first assessed among population-year variation in NDVI curves to test direct and indirect (i.e. through body mass) effects of changes in plant phenology on overwinter survival of fawns. In most previous studies (see table 1 for a review), ecologists have either used *a priori* summary statistics of NDVI. Unfortunately, this approach has led to the use of only a few variables to define the growing season in any ecosystem; thus to more completely assess vegetation phenology, we proposed a new approach to identify the key periods along the NDVI curve. Instead of defining these periods *a priori*, our approach is based on a multivariate functional analysis of variation in observed NDVI curves.

We used FPCA, a type of functional data analysis (FDA) to analyse among-population and among-year variation in NDVI curves. FDA is specifically designed to characterize information in multivariate time series [51]. FPCA techniques are relatively recent [51] and surprisingly rarely used in ecology and remote sensing (but see [81]) even if they offer a very powerful way to analyse temporal ecological data such as NDVI time series. FPCA was applied to NDVI curves to identify spatio-temporal patterns of vegetation changes. While *a priori* defined metrics estimated from NDVI data have occasionally been analysed using principal components analysis (PCA) [37], standard PCA is not optimal for time-series data. In PCA, weeks would be considered as independent vectors of values, whereas functional PCA (FPCA) explicitly accounts for the chronology of weeks by treating the statistical unit as the individual NDVI curve. This ensures that the patterns identified by FPCA are constrained to be temporal trends within the growing period (i.e. portions of the curve) and not due to few independent NDVI values. FPCA produces eigenvalues (measuring variation explained by each dimension) and principal component scores for sampling units (summarizing similarities among NDVI curves). However, eigenvectors are replaced by eigenfunctions (harmonics) that show the major functional variations associated to each dimension.

To facilitate the application of FPCA by ecologists and remote sensing scientists, we have provided in the electronic supplementary materials the data and the full R code (based on the fda package) to reproduce the analysis performed in the paper. As these methods are poorly known in ecology and remote sensing, we have also provided an expanded description of the mathematical theory, but the reader could consult the original books [51,82] for additional information.

Sampling units (population-years) were partitioned using the *k*-means algorithm applied on the first two principal component scores. We computed the Calinski and Harabasz criterion for partitions between two and 10 groups, and select the optimal number of clusters that maximizes the criterion. We also computed the amount of variation in the first two principal component scores (NDVI curves) that were explained by space (i.e. population) and time (year). This allowed us to understand which source of variation contributed most to differences in growing season dynamics. We then used principal component scores in subsequent analyses as explanatory variables of mule deer fawn mass and survival.

### (e) PRISM weather data

We characterized winter (1 November to 31 March) weather conditions using 4 km gridded PRISM observations of minimum monthly temperature and total monthly precipitation from 1995 to 2011 [83] (available from http://www.prism.oregonstate.edu). Temperature and precipitation data were averaged across the winter range for each population, and then summed (averaged) across months for precipitation (temperature) to produce climate covariates that represented measures of winter severity, respectively. We produced variables for early winter (November–December) and late winter (January–March) for both precipitation and temperature. These variables were highly correlated (*r* > 0.4); thus we selected the variable with the highest first-order correlation to our response variable, overwinter survival of fawns, as our winter severity index.

### (f) Environmental effects on body mass and overwinter survival of fawns

We estimated population- and year-specific estimates of overwinter fawn survival (from 16 December to 1 June) using staggered Kaplan–Meier non-parametric survival models. We then employed path analysis [52] to test the population-level effects of body mass and winter weather, and to tease apart the direct from the indirect effects (through fawn body mass, see figure 3) of key periods of NDVI on overwinter survival. For the path analysis, we transformed our response variable with an empirical logit function [84] because average survival for each population-year is a proportion bounded between 0 and 1 [85]. We used mass of female fawns in December to measure the cohort quality of the birth year [86] and eliminate the effect of sexual size dimorphism [63]. A first, indirect, mechanistic link between environmental conditions early in life and overwinter survival could be that variation plant phenology and nutritional quality affects the body development of fawns, which in turn, drives overwinter survival. An alternative could be that variation in plant phenology is directly related to overwinter survival as a result of the availability and quality of winter forage. Because winter precipitation was recorded in November–December at the same time as the weighing of fawns, we could not test for an indirect effect of winter precipitation through body mass on overwinter survival. Our model included a population effect entered as a random factor on the intercept to account for the repeated measurements of overwinter survival in different years within a population.

We used a Bayesian framework to fit the path analyses to our data [87]. We used non-informative normal (mean of 0 and a s.d. of 100) and uniform (range between 0 and 100) priors for the regression coefficients and variance parameters, respectively. Using JAGS [88], we generated 50 000 samples from Monte Carlo Markov chains to build the posterior distributions of estimated parameters after discarding the first 5000 iterations as a burn in. We checked convergence graphically and based on Gelman's statistics [87]. Estimated parameters were given by computing the mean of the posterior distribution, and the 2.5th and 97.5th percentiles of the distribution provided its 95% credibility interval. We considered a variable as statistically significant if the credibility interval of its posterior distribution excluded 0. We assessed the fit of the model by computing the squared correlation coefficient between observed and predicted values [89]. Finally, to compare the relative effect sizes of the explanatory variables on overwinter survival, we replicated the analyses using standardized coefficients.

## 3. Results

### (a) Functional analysis of Normalized Difference Vegetation Index curves

FPCA of NDVI data led to the identification of two independent eigenfunctions (hereafter FPCA components), which reflected contrasting patterns of plant phenology in spring and autumn. Both FPCA components corresponded to continua of increasing NDVI intensity, in early and late growing seasons, and were used as explanatory variables of overwinter survival of mule deer fawns.

The first FPCA component described the late season phenology, after peak value, and accounted for 48.9% of the total variation in NDVI curves. The second FPCA component represented the early season phenology and accounted for approximately half as much variation as the first FPCA component (27%; figure 1). FPCA components can be interpreted as the amount of deviation from the overall average NDVI curve in terms of overall primary productivity at different times within the growing season. For example, high FPCA component 1 scores mean both high primary productivity in open habitats in autumn, but also a longer autumn growing season compared to lower FPCA component 1 scores (figure 1*a*,*c*). Similarly, positive values of FPCA component 2 reflect both higher spring primary productivity and early onset of plant growth (e.g. figure 1*b*,*c*; type 4 dark green).

**Figure 2.**
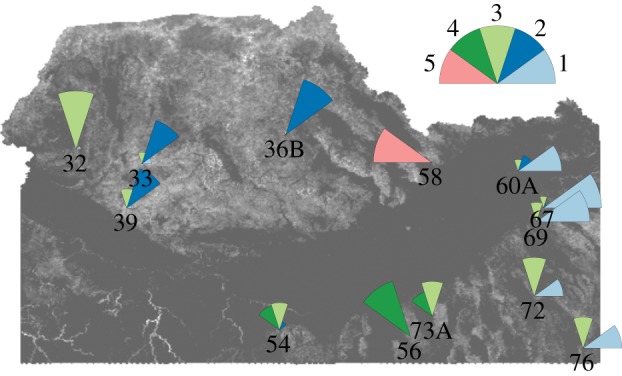
Distribution of the five NDVI typologies shown in figure 1, with corresponding colours (inset) across the 13 mule deer populations (GMUs) in Idaho, USA, from 1998 to 2011. The size of the pie wedge is proportional to the frequency of occurrence of each NDVI typology within that mule deer population. For example, population 56 had all but one population-year occurring in NDVI typology 4 (figure 1) indicating low primary productivity during spring but higher during autumn.

Combining both continua in a factorial plane allowed us to distinguish five NDVI types of curve in reference to the overall mean trend (figure 1*c*). For example, NDVI in autumn was close to the average for the NDVI curve type 2 (dark blue, figure 1*c*), but NDVI in spring was the lowest of all curve types in figure 1*c*. Conversely, NDVI curve type 3 (light green, figure 1*c*) has NDVI values above average in both spring and autumn. The NDVI curve type 1 (light blue, figure 1*c*) has the highest NDVI in autumn, while NDVI curve type 5 (red, figure 1*c*) had lowest autumn productivity. Generally, a given population displayed one NDVI curve type, with some extreme values belonging to a different type (figure 2, see also the electronic supplemental material, figure S1). Decomposition of the among-population and among-year variance in NDVI curves in fact shows that most (73.8%) of the observed variation in NDVI curves was accounted for by population (i.e. spatial variation), and much less (20.8%) by annual variation within a population, with a high degree of synchrony between populations within a year (only 5.4% of the variation in NDVI curves is unexplained). This suggests that the five NDVI types we identified (figure 1) strongly reflect the distribution of ecotypes and vegetation characteristics among populations (figure 2).

### (b) Environmental effects on body mass and overwinter survival of fawns

**Figure 3.**
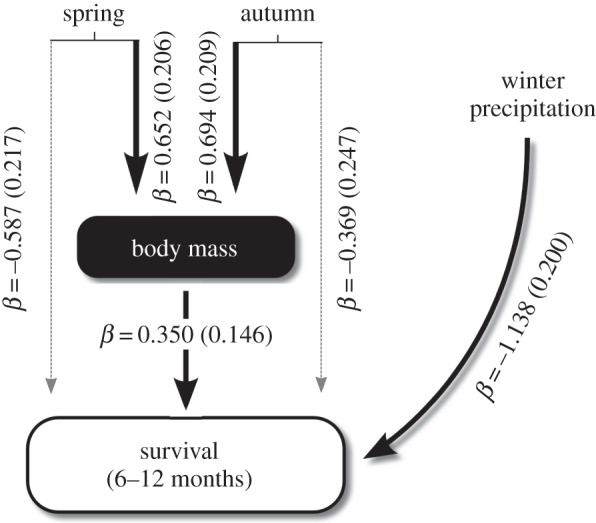
Hierarchical Bayesian path analysis of the effects of spring and autumn growing season functional components (from figure 1) and winter precipitation on mule deer fawn body mass and overwinter survival from 1998 to 2011 in Idaho, USA. This model explained 44.5% of the variation in survival. Beta coefficients and their s.d. are shown, with solid lines indicating the indirect effects of NDVI on survival through their effects on body mass, and dashed lines indicate the direct effects of NDVI on survival.

**Figure 4.**
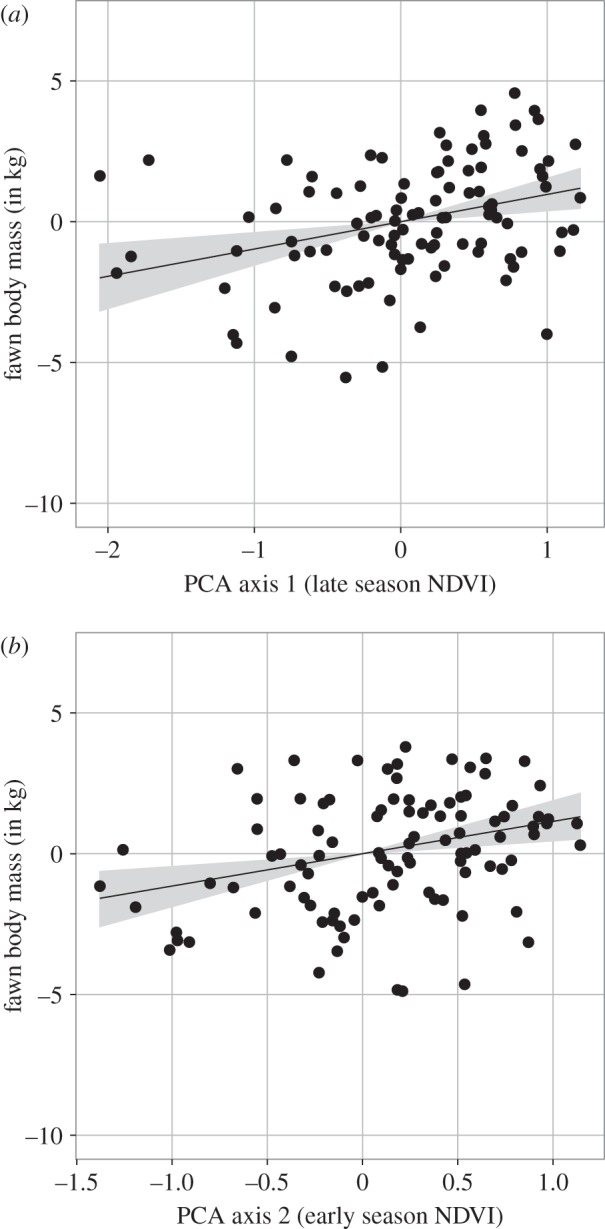
Results of hierarchical Bayesian path analysis showing the standardized direct effects of (*a*) FPCA component 1 from the functional analysis (autumn NDVI) and (*b*) FPCA component 2 (Spring NDVI) on body mass (kg) of mule deer fawns in Idaho, USA, from 1998 to 2011.

The average body mass of female fawns in December was 34.0 kg (s.e. = 2.55). In agreement with our hypothesis, body mass of six-month-old fawns was positively related to NDVI in both spring and autumn (figures 3 and 4). From the estimated standardized regression coefficients, the effect of NDVI in autumn (FPCA component 1) on autumn body mass of fawns (standardized *β* = 0.694, s.e. = 0.209) was greater than the effect of NDVI in spring (FPCA component 2; standardized *β* = 0.652, s.e. = 0.206). FPCA component in the autumn explained more variance in body mass than traditional estimates of phenology such as, start, end or peak date of growing season (electronic supplemental material, table S3). The autumn was thus of more importance to the body development of mule deer fawns at the onset of winter than spring (figures 3 and 4).

**Figure 5.**
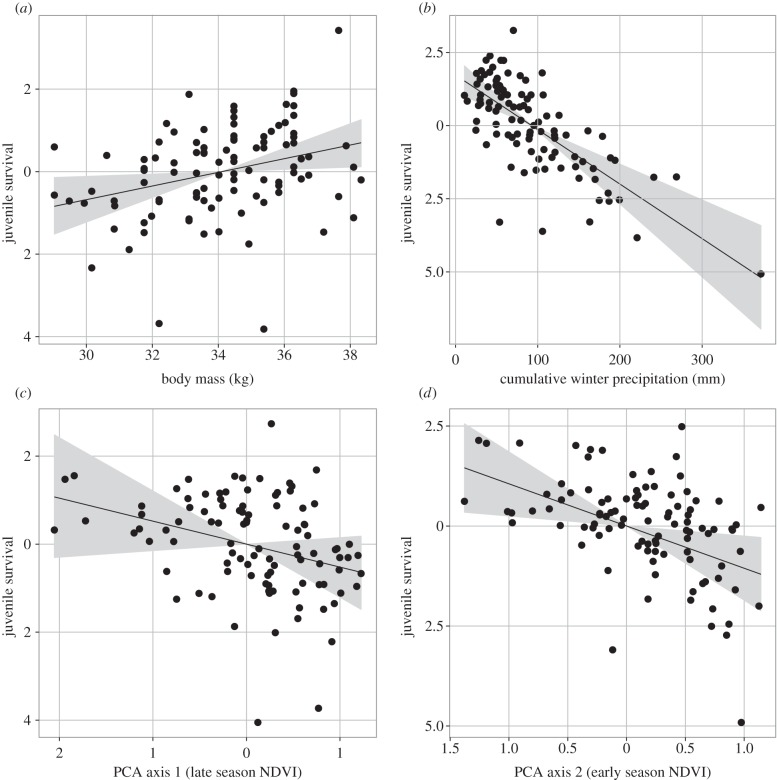
Results of hierarchical Bayesian path analysis showing standardized direct effects of (*a*) body mass (kg), (*b*) cumulative winter precipitation (in mm) and (*c*) FPCA component 1 from the functional analysis (autumn NDVI) and (*d*) FPCA component 2 (spring NDVI) on the overwinter survival of mule deer fawns in Idaho, USA, from 1998 to 2011.

The annual overwinter survival of mule deer fawns averaged 0.55 (s.e. = 0.24, range = 0–0.94) across populations. Our best model accounted for 44.5% of the observed variation in overwinter survival, including the additive effects of autumn body mass of female fawns, early winter precipitation and of spring and autumn NDVI. As expected, when mean body mass reflects the average demographic performance of a given cohort, the annual overwinter survival of fawns was associated positively with the mean cohort body mass in late autumn (figures 3 and 5*a*). Total precipitation during early winter from November to December (ranging from 11 to 372 mm) was associated with decreased fawn survival (figures 3 and 5*b*). Once the effect of body mass and winter precipitations were accounted for, spring had negative impacts on the overwinter survival of fawns (figures 3 and 5*d*), so that survival was lower with higher NDVI during the spring plant growth season. Autumn was not significantly related to overwinter survival beyond the positive effect on body mass. Winter precipitation has the greatest effect size on overwinter survival of fawns (standardized *β* = −1.138, s.d. = 0.200), followed by spring (standardized *β* = −0.587, s.d. = 0.217) and autumn (standardized *β* = −0.369, s.d. = 0.247), while fawn body mass in autumn has the smallest relative effect size (standardized *β* = 0.350, s.d. = 0.146). The observed relationships between environmental conditions and overwinter survival of fawns differed slightly among populations but differences were not statistically supported (electronic supplementary material, figure S2).

## 4. Discussion

Our results linked variation in observed plant phenology to body mass and survival of juvenile mule deer during winter across populations and years, demonstrating the benefits of connecting remote sensing and biological information to understand consequences of environmental change on biodiversity. We used a new statistical approach to identify plant phenology from NDVI curves encompassing the entire growing season. Previous studies have reported effects of plant phenology on body mass and demographic parameters in several species of mammals and birds (see table 1 for a review). However, all these studies but one [40] were based on *a priori* defined metrics mostly focusing on indices of spring phenology; thus spring metrics appear to explain population parameters, but the relative role of late plant growth season has rarely been investigated. Our approach provides a compelling example and motivation for functional analysis of remote-sensing-derived measures of plant growth as a first step to help identify plant phenological periods most affecting population dynamics of animals.

Our results emphasized that the relative role of spring versus autumn phenology is unclear for ungulate species adapted to more arid environments. By defining the periods *a posteriori*, we found that mule deer fawns survived better in populations with higher NDVI during autumn, and thus longer autumn growing seasons. The effect size of autumn NDVI was stronger than the effect size of spring NDVI for predicting six-month-old body mass. Body mass was positively related to overwinter survival, but precipitation during early winter decreased survival with an effect size almost three times as strong as early winter body mass, similar to other studies of winter ungulate survival [63,90,91]. Previous studies on large herbivores reported an effect of the preceding winter conditions when the juvenile was *in utero* [37,40,70,92] or an effect of spring conditions [37] on body mass. The patterns of variation in NDVI curves translated to spatial variation in plant growth during autumn, and hence mule deer body mass and survival. First, we found almost twice as much variation in the NDVI curves occurred in the autumn (FPCA component 1, figure 1*a*) compared with spring (FPCA component 2, figure 1*a*). Thus, plant phenology during the autumn was more variable than spring in our semi-arid system. Second, we found almost three times the variation in NDVI curves was explained by spatial variation among populations in a given year compared with among-year variation. The high proportion of the variance explained among populations indicates that variation among NDVI curves within a population was consistent from year to year and also synchronous between units within a year. These patterns of stronger variation during autumn (versus spring) and among populations (versus among years) contributed to autumn NDVI having double the effect size on body mass, and hence survival. Thus, the most variable period of the growing season (e.g. autumn) had the strongest effect size on mass and survival. These results mirror results from studies of just the spatial variance in survival [93] and suggest that plant phenology may also synchronize population dynamics. With the recent focus on autumn nutrition of elk [53], however, many ungulate managers in North America are focusing increasingly on autumn nutrition. Our results emphasize that, at least for large herbivores, focusing *a priori* on just one season, spring or autumn, without explicit consideration of the spatio-temporal variation in the entire curve of plant phenology could be misleading.

Forage availability for large herbivores varied by vegetation cover type, precipitation and temperature during the growing season [55,94]. Increased rainfall in summer, reflected in increased NDVI in autumn, will promote growth of forbs [94], a highly selected forage for mule deer [94,95], and can promote new growth in autumn germinating annual graminoids (e.g. cheatgrass, *Bromus tectorum*) and delay senescence, prolonging access to higher quality forage [14]. Increased summer–autumn nutrition improved calf and adult female survival, fecundity rates and age of first reproduction in captive elk [53]. Rainfall during the growing season also increases quality and quantity of winter forage [94], which increases survival of fawns and adult female mule deer [58]. Tollefson *et al*. [57] showed that summer forage has the greatest impact on mule deer juvenile survival and overall population growth rate in a penned experiment in eastern Washington, USA. In our study area, effects of climate and plant phenology certainly varied across our southeast to northwest gradient (electronic supplementary material), but will require individual-level analyses of single radiocollared mule deer to most clearly separate out local influences on overwinter survival. Therefore, especially in arid or semi-arid systems, we expect that future studies will identify strong signatures of autumn NDVI and climate on demographic parameters of large herbivore populations, similar to our results.

One obvious difference between our arid study system and previous studies of NDVI and large herbivores is that NDVI curves were not a classic bell shape. Instead, plants in open-habitats had a left-skewed growth curve, with a rapid green-up in spring, but then a long right tail in the NDVI distribution, and, occasionally, secondary growth peaks in late summer and autumn (e.g. figure 1*c*). Most other studies that examined NDVI curves found more symmetrical shapes, with a rapid plant green-up and senescence [37,96]. However, Martinez-Jauregui *et al*. [25] found the classic bell-shaped NDVI curve for Norwegian and Scottish red deer (*C. elaphus*), but a similarly earlier and flatter NDVI curve in southern Spain. We believe our right-skewed autumn growing season dynamics may be characteristic of arid or semi-arid systems where precipitation and growing seasons cease during summer. Nonetheless, the variability among studies in the shape of the NDVI curves emphasizes the importance of identifying key periods of the growing season *a posteriori*.

One unexpected result from our study was the negative direct effects of spring NDVI on overwinter survival of mule deer fawns, in contrast to the stronger positive effect of both spring and autumn NDVI on body mass, and of body mass on overwinter fawn survival. There could be several competing explanations for this puzzling result. First, despite the power of path analysis at disentangling complex relationships [52], there could still remain some confounding effects of body mass or winter severity. Although we attempted to control for spatial variability with random effects of study site, there could also be negative covariance between winter severity, which, because spring NDVI is correlated to winter severity of the preceding winter [50], could lead to negative correlation between spring NDVI and subsequent winter severity. The effect of this general relationship may downscale to study site differently if snow depth passes a threshold where few fawns survive regardless of mass, as is the case sporadically in some of our higher elevation study sites [96–98] that typically display the most productive NDVI curve types. Mysterud & Austrheim [97] provide a very plausible explanation based on how the negative effect of a later spring (axis 2) will increase winter survival through prolonging access to high-quality forage. Alternatively, viability selection operating on mule deer cohorts may explain this pattern [99]. Counterintuitively, if good spring growing conditions enhance summer survival, a large proportion of the cohort will survive until the onset of the winter, including frail [100] individuals that would experience increased mortality during winter [98], and the opposite during harsh springs. As individual early mortality in populations of large herbivores is tightly linked with maternal condition [66], fawns surviving to the winter will be mostly high-quality fawns enjoying high maternal condition. Those fawns would thus be expected to be robust enough to survive winter. Bishop *et al*. [58] suggested this exact viability selection process for mule deer fawns in Colorado, supporting our interpretation of this counterintuitive spring NDVI effect. Viability selection could also be compounded through the interaction between winter severity and the preponderance of predator-caused mortality in winter [63]. There might also be negative covariance between neonate and overwinter survival [58], driven as we suggest here by different spring and autumn phenology patterns. Regardless, many plausible biological processes exist to explain the effect of early season plant growth on winter survival of fawns.

Functional analysis provides a powerful approach to identify the key periods of the growing season from remote sensing data and to assess their differential effects on life-history traits. Our functional analysis applied to year- and population-specific NDVI curves allowed us to identify two distinct components of variation that corresponded closely to contrasting spring and autumn phenology. Of course, many remote sensing studies have used NDVI for decades to examine differences in spring and autumn phenology [6]. Yet, despite the primacy of multivariate approaches in remote sensing, only a few studies have used even standard PCA to examine spatial trends in NDVI [101] or identify NDVI anomalies [102]. Functional analysis allowed us to identify phenological patterns *a posteriori* and to summarize NDVI curves into only two independent components instead of 5–12 *a priori* defined metrics that are strongly correlated (table 1). Moreover, our FPCA axes explained variation similarly or better than pre-defined parameters based on previous studies (e.g. axis 1 versus senescence date; electronic supplementary material, table S3). Functional analysis provides a novel and powerful approach for studies of the ecological effects of plant phenology, and arose out of the productive collaboration between remote sensing scientists and ecologists. We anticipate the benefits of functional analyses to extend far beyond NDVI, to ecological analyses of variation in the other remotely sensed vegetation indices (e.g. fPAR, EVI), MODIS snow and temperature datasets, and aquatic measures such as sea surface temperature, chlorophyll and other important ecological drivers.

In conclusion, in large parts of the world that are semi-arid or deserts, our results strongly show that it may not be just spring phenology that matters to ungulate population dynamics. Our new approach using functional analysis of the entire NDVI curve provides a powerful method to identify first key periods within the growing season and then disentangle their respective roles on demographic traits when combined with hierarchical path analysis. Our approach thus allowed us to determine the most likely pathways by which plant growth influenced mule deer overwinter survival of fawns. Finally, and perhaps most importantly, we demonstrated a novel approach to first identify different temporal components of remote sensing datasets that are the key drivers of large-scale population responses, aiding the broad objective of enhancing our ability to monitor responses of biodiversity to environmental change at global scales.

## Notes

Mule deer capture and handling methods were approved by IDFG (Animal Care and Use Committee, IDFG Wildlife Health Laboratory) and University of Montana IACUC (protocol no. 02-11MHCFC-031811).
